# Editorial: The neuroendocrine female brain: from normal reproductive function to disease

**DOI:** 10.3389/fnins.2023.1243349

**Published:** 2023-07-13

**Authors:** Mauro S. B. Silva, Victor M. Navarro, Konstantina Chachlaki

**Affiliations:** ^1^Brigham and Women's Hospital and Harvard Medical School, Boston, MA, United States; ^2^Université de Lille, Inserm, CHU Lille, Laboratory of Development and Plasticity of the Neuroendocrine Brain, Lille Neuroscience and Cognition, UMR-S 1172, Lille, France; ^3^FHU 1000 Days for Health, School of Medicine, Lille, France; ^4^University Research Institute of Child Health and Precision Medicine, National and Kapodistrian University of Athens, “Aghia Sophia” Children's Hospital, Athens, Greece

**Keywords:** neuroendocrinology and reproduction, female health, female reproduction, neuroendocrine system, GnRH, neurons and networks

Reproductive function is often associated only with the gonads, both male and female, which work together for the sole purpose of procreating, according to lay perception and discussion. Beyond this simplistic view, reproduction is an umbrella term covering a range of functions, such as sexual performance, its influence on social behaviors, their impact on biological rhythms, and offspring care. These examples clearly illustrate the fact that the brain is central in how reproduction operates to guarantee the enduring survival of a species. Neural communication is largely supported by peripheral hormones, which keep regulatory mechanisms in check, while neurons and glial cells secrete neurohormones acting locally to maintain homeostasis. Thus, neuroendocrine systems influence physiological and behavioral states, such as reproductive functioning, with the refinement of sex being one of the strongest forces shaping these systems differently in men and women.

We encounter intrinsic challenges in studying neuroendocrine systems due to the brain's heterogeneity and the complex organization of hormone-sensitive neurons in the hypothalamus. In female individuals, the mechanisms behind this complexity seem to be more diverse, with connections (for instance) to the reproductive cycle, metabolic demands, and female-specific behaviors (Goodman et al., 2022). This diversity, however, should not preclude ongoing and future scientific endeavors, as it creates a unique opportunity to uncover the intricate nature of the female brain. This Research Topic ventures to deliver novel insights that develop our understanding of the molecular and cellular mechanisms underpinning the neuroendocrine female brain under healthy and pathological conditions. We present a collection of original research articles and reviews highlighting the ways in which neuroendocrine circuits govern female reproductive life.

Gonadotropin-releasing hormone (GnRH) neurons are considered to be the pacemaker cells in the hypothalamus, driving the motion of reproductive cycles in all vertebrates. These neurons orchestrate the secretion of gonadotrophins, such as luteinizing hormone (LH) and follicle-stimulating hormone (FSH), by the pituitary gland in both sexes (Herbison, 2014; Plant, 2015; Zeleznik and Plant, 2015). While both sexes mostly exhibit episodic release of LH in a pulse-like manner, females also display a robust pre-ovulatory LH surge, which is critical to ovulation and fertility. Kauffman from the University of California, San Diego, presents a well-constructed, comprehensive review of the neuronal and endocrine components driving the LH surge. For instance, estradiol acts in upstream components to GnRH neurons known as kisspeptin neurons to fully establish surge induction. Kisspeptin neurons are central in promoting GnRH pulse generation, which drives the episodic LH release in order to maintain reproductive competence (Goodman et al., 2022). These cells are also under an inhibitory tone regulated by opioids, which are molecules well-known for their role in analgesia and substance abuse. On this subject, the contribution by Uenoyama et al. to this compilation presents a historical and mechanistic review of the role of endogenous opioids in kisspeptinergic function, feedback regulation, and female reproduction.

It is dramatically clear that the mechanisms controlling female sexual behaviors are far less well understood than those controlling these behaviors in males. This is also reflected in the scarcity of therapies and hypothesis-driven interventions targeting treatment of female sexual dysfunction. Semple et al. explore this topic by interrogating the function of melanocortin signaling, which plays a role in inducing sexual behavior in female mice, in transcription factor single-minded homolog 1 (Sim1) and oxytocin receptor (OTX) neurons using a range of transgenic technology and behavioral testing. An important dimorphism in social behaviors has been observed in the study of aggression in mammals, in which males usually display increased aggression to guarantee their hierarchical status while females increase their aggression levels during maternal care and lactation periods. Oliveira and Bakker review different animal models of maternal and non-maternal female aggression and present novel insights into the neuroendocrine circuitry underlying normal and pathological levels of female aggression. In this Research Topic, we additionally present an original research article in which Oliveira et al. explore the functional role of oxytocin and arginine vasopressin signaling in GABA neurons of the central amygdala in the escalation of female aggression.

This Research Topic also explores how biological rhythms are entrenched in the female brain, as these biological clocks strongly influence health and well-being in women working under constant changes in daily shifts, for instance. The suprachiasmatic nucleus (SCN) of the hypothalamus is considered to be the brain's circadian master clock, expressing genes and neurohormones that govern biological rhythms (Hastings et al., 2014). Using various transgenic approaches, Tonsfeldt et al. dissect the role of the core clock gene *Bmal1* in different neuroendocrine SCN neurons in female mice, with a substantial focus on fertility. The SCN is also under the control of gonadal steroid hormones, such as testosterone, in both sexes. However, polycystic ovary syndrome (PCOS) is the most common endocrine female disorder leading to infertility worldwide, in which testosterone levels are commonly higher than in healthy women (Azziz et al., 2016). Jamieson et al. explore important evidence relating to this topic to show how hyperandrogenemia disrupts neuroendocrine SCN neurons in a pre-clinical PCOS mouse model. Despite great concern regarding disruptions to the biological clock, there is a lack of knowledge of how sleep pattern disturbances affect women's health during different periods of their reproductive life. To fill this knowledge gap, Liu et al. aim to identify sleep-related factors that influence the risk of stroke during menopause, when reproductive hormones undergo dramatic changes in women.

In collating the articles for this Research Topic, our goal was to dive into the unexplored terrain of the female neuroendocrine brain and its implications for health and science. We aspire to present novel pathways for this research focus in order to leverage current knowledge in the field and to advance toward a better understanding of the fascinating mysteries of the female brain in terms of its role in control of reproduction.

## Author contributions

MS conceived and wrote the manuscript with great scientific input from VN and KC. All authors contributed to the article and approved the submitted version.
